# Potential involvement of adiponectin in obesity-associated erosive esophagitis

**DOI:** 10.3164/jcbn.17-65

**Published:** 2020-07-16

**Authors:** Chung Hyun Tae, Hye-Kyung Jung, Seong-Eun Kim, Sung-Ae Jung, Sun Ha Jee

**Affiliations:** 1 Department of Internal Medicine, College of Medicine, Ewha Womans University, 1071 Anyangcheon-ro, Yangcheon-gu, Seoul 07985, Korea; 2 Department of Epidemiology and Health Promotion Institute for Health Promotion, Graduate School of Public Health, Yonsei University, 50 Yonsei-ro, Seodaemun-gu, Seoul 03722, Korea

**Keywords:** adiponectin, gastroesophageal reflux disease, obesity, gender difference

## Abstract

A strong causal relationship between obesity and erosive esophagitis has been proposed. Obesity may affect the pathogenesis of erosive esophagitis through adipokines as well as acid reflux. We evaluated the involvement of adiponectin in obesity-associated erosive esophagitis. In total, 1,902 patients who underwent endoscopy during medical check-ups were selected for study. Variables including the body mass index (BMI) and adiponectin level were compared between subjects with erosive esophagitis and normal controls. The subjects were classified by quartiles (Qs) of adiponectin level. Q4 was the reference group. The median adiponectin level differed by gender (men, 5.3 µg/ml vs women, 9.3 µg/ml; *p*<0.001). As the severity of erosive esophagitis increased in men, the BMI increased (*p*<0.001) while the adiponectin level decreased (*p* = 0.026). The multivariate odds ratio for erosive esophagitis was 1.79 for Q1, 1.73 for Q2, 2.34 for obesity, and 27.40 for hiatal hernia in men. When classified by obesity, the multivariate odds ratio for erosive esophagitis was 1.94 for Q1, 2.10 for Q2, and 18.47 for hiatal hernia only in obese men. In women, there were no trends in BMI, adiponectin levels, or severity of erosive esophagitis. We demonstrated that low adiponectin levels are involved in obesity-associated erosive esophagitis in men but not women.

## Introduction

Gastroesophageal reflux disease (GERD) may have different causative factors between obese and lean individuals.^(1)^ Although a defective esophagogastric barrier is usually found in non-obese individuals with GERD, a strong causal link between obesity and erosive esophagitis (EE) has been reported.^(1,2)^ Meta-analyses have shown a positive correlation between obesity and GERD.^(3–5)^ In addition, weight reduction through lifestyle modification or diet intervention leads to less acid reflux and symptom relief in patients with GERD.^(6,7)^ Various mechanisms have been proposed for the close relationship between GERD and obesity. This relationship has traditionally been linked to several mechanical and motility changes in the gastroesophageal anatomy.^(8)^ Studies that have used esophageal manometry and pH monitoring have revealed that obese individuals have esophageal motor disorders such as ineffective esophageal motility, a nutcracker esophagus, a hypertensive lower esophageal sphincter (LES), diffuse esophageal spasm, a hypotensive LES, and transient relaxation of the LES.^(9–14)^ The presence of a hiatal hernia and increased intragastric pressure secondary to increased intra-abdominal pressure are associated with GERD in obese subjects.^(14,15)^ However, the precise pathophysiological link between obesity and GERD has not been fully elucidated.

Recently, there has been growing interest in adiponectin, an adipokine secreted from adipose tissue. One study reported that adiponectin levels were significantly lower in obese subjects than in non-obese subjects.^(16)^ Another study found that increases in adiponectin levels were accompanied by reductions in the body mass index (BMI).^(17)^ Therefore, it has been suggested that adiponectin might play a biologically protective role in obesity-associated disorders.^(17)^ A few studies have addressed the role of adiponectin in GERD. One study revealed that low adiponectin levels were associated with GERD severity scores as defined by videoesophagography in obese subjects, and another study showed a reverse correlation between adiponectin levels and the severity of EE in men.^(18,19)^ In a recent study, the adiponectin levels of 23 men with EE were lower than those of 8 men without EE; this finding was not detected in women.^(20)^ However, these studies had some methodological limitations such as only included men and applied only univariate analyses.

We investigated the potential link between adiponectin deficiency and obesity-associated EE in a large representative population of both men and women, and applied both univariate and multivariate analyses.

## Patients and Methods

### Subjects

**Fig. 1:**
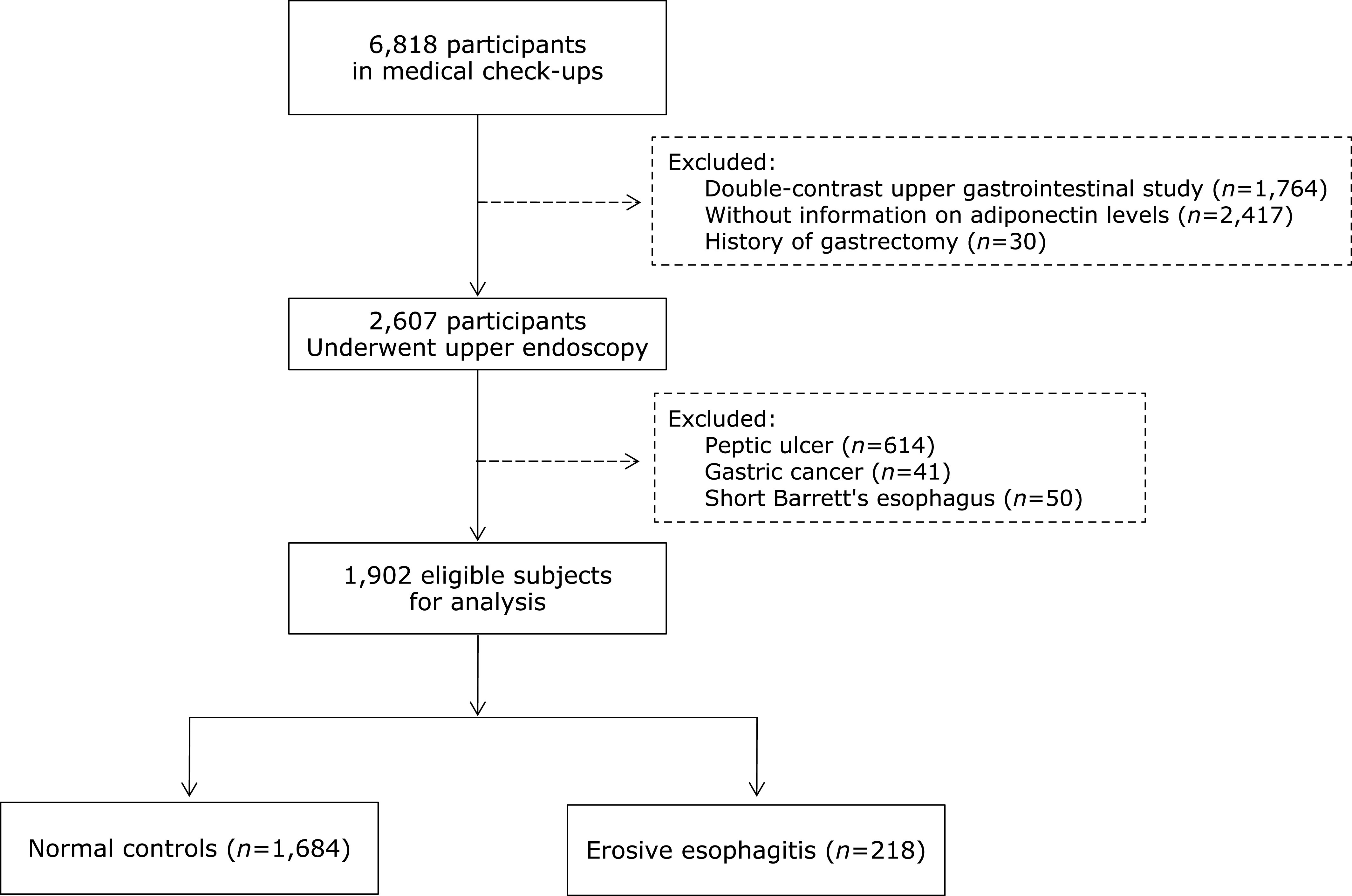
Flow chart of study population selection.

The subjects were from a cohort of voluntary participants undergoing medical check-ups at the health-promotion center of a tertiary hospital. Of 6,818 participants, 1,902 eligible subjects (1,122 men and 780 women) who met the following criteria were enrolled: those who underwent upper endoscopy, blood chemistry tests including measurement of adiponectin levels, and measurement of anthropometric parameters such as BMI and waist circumference. We excluded subjects who had undergone a double-contrast upper gastrointestinal study instead of upper endoscopy (*n* = 1,764), those without information on adiponectin levels (*n* = 2,417), those with a history of gastrectomy (*n* = 30), and those who had been diagnosed with peptic ulcer (*n* = 614), gastric cancer (*n* = 41), or short Barrett’s esophagus (*n* = 50) (Fig. 1). The present study protocol was reviewed and approved by the Institutional Review Board of Severance (IRB No. 4-2005-0197). Informed consent was submitted by all subjects when they were enrolled.

### Upper endoscopy examination and endoscopic severity of EE

All endoscopic procedures were performed by two board-certified gastroenterologists. In the diagnosis of EE and determination of its severity, each gastroenterologist independently interpreted the findings based on the Los Angeles (LA) classification and assigned one of the following grades: grade LA-A, one or more mucosal breaks confined to the mucosal folds (each <5 mm); grade LA-B, at least one mucosal break >5 mm long confined to the mucosal folds; grade LA-C, at least one mucosal break continuing between the tops of two or more mucosal folds but involving <75% of the esophageal circumference; and grade LA-D, one or more mucosal breaks involving at least 75% of the esophageal circumference. Subjects with minimal change in the esophagogastric junction were considered normal controls and were not considered to have EE because of interobserver variability. A hiatal hernia was diagnosed if the distance between the gastroesophageal junction and the diaphragmatic hiatus was 2 cm or more.

### Questionnaire and anthropometry

This study was conducted according to a standard protocol. All subjects were asked to complete a structured questionnaire. It contained questions regarding the subjects’ smoking status (current smoker or nonsmoker), alcohol consumption (any type of beverage, average frequency, amount), and medical history (hypertension, diabetes mellitus), and medications (non-steroidal anti-inflammatory drug, proton pump inhibitor, histamine 2 blocker, statin, thiazolidinedione). We calculated the amount of ethanol intake per week by multiplying the frequency of consumption of each beverage by the alcohol content of the specified portion size. Those who consumed >140 g alcohol per week were defined as heavy drinkers. Anthropometric measurements such as weight, height, and waist circumference were also obtained. We calculated the BMI as the ratio of weight (kg) divided by height (m) in square meters.

### Blood chemistry and adiponectin assay

Fasting morning blood samples were collected, stored at –80°C within 2 h, and saved until analysis. The levels of total cholesterol (TC), triglyceride (TG), high-density lipoprotein cholesterol (HDL-C), low-density lipoprotein cholesterol (LDL-C), and fasting venous glucose were measured with a Hitachi 7600 analyzer (Hitachi Ltd., Tokyo, Japan). Serum levels of insulin and high-sensitivity C reactive protein (hs-CRP) were analyzed by solid-phase two-site chemiluminescent immunometric assay using an IMMULITE 2000 (Diamond Diagnostics, Holliston, MA). Serum levels of adiponectin were measured using an enzyme-linked immunosorbent assay kit (Mesdia Co., Ltd., Seoul, Korea). Intra- and inter-assay controls were performed in accordance with the procedures of the Korean Association of Laboratory Quality Control and were 6.3% to7.4% and 4.5% to 8.6%, respectively.^(21)^

The homeostasis model assessment of insulin resistance (HOMA-IR) was calculated by multiplying the fasting serum concentrations of insulin (µU/ml) and glucose (mmol/L) and dividing the result by 22.5.^(22)^

According to the Modified National Cholesterol Education Program Adult Treatment Panel III criteria, metabolic syndrome was defined as the presence of at least three of the following five abnormalities: a high waist circumference (≥90 cm in men and ≥80 cm in women); high TG levels (≥150 mg/dl); low HDL-C levels (≤40 mg/dl in men and ≤50 mg/dl in women); high blood pressure (systolic ≥130 mmHg, diastolic ≥85 mmHg) or treatment for previously diagnosed hypertension; and elevated fasting serum level of glucose (≥110 mg/dl) or taking medication for diabetes mellitus.^(23)^

### Statistical analysis

The Statistical Package for the Social Sciences ver. 20.0 (SPSS Inc., Chicago, IL) was used for data entry and statistical analyses. The results are presented as mean ± SD or *n* (%) for categorical variables. Student’s *t* test was performed to compare the mean results of normally distributed continuous variables, while the chi-square test was used for categorical variables. To measure the strengths of association between adiponectin and other clinical variables, such as age, BMI, waist circumference, HOMA-IR, hs-CRP, TC, TG, HDL-C, and LDL-C, we calculated using Spearman correlation coefficients. Adiponectin levels showed a skewed distribution and gender-related differences. Therefore, we expressed the median of adiponectin levels and categorized into quartiles (Qs) according to gender. Three cut-off points were used: 3.7, 5.3, and 7.8 µg/ml for men and 6.2, 9.3, and 13.2 µg/ml for women. In all models, Q4 of the adiponectin level was considered the reference group.

To calculate the chi-square for linearity of numerical values in following contingencies such as both severity of EE and BMI, both severity of EE and adiponectin levels, and both Qs of the adiponectin levels and prevalence of EE, the Mantel-Haenszel linear-by-linear association was performed. Multivariate analyses were adjusted for age and four statistically significant variables: the Qs of adiponectin level, obesity, hiatal hernia, and metabolic syndrome. These four variables were significantly different in both normal controls and subjects with EE in univariate analyses and therefore were subjected multivariate analyses. Meanwhile, the waist circumference, HOMA-IR, and TG levels were significant factors in univariate analyses. However, their variance-inflated factors exceeded 10 in the multivariate model; therefore, they were not included. We considered that obesity might be a major confounding factor in the association between adiponectin and EE. To clarify the independent predictors of obesity-associated EE, we performed multivariate analyses by dividing the subjects into obese and non-obese subjects. Obesity was defined as a BMI of 25 kg/m^2^ or higher based on Asia-Pacific criteria.^(24)^ The odds ratio (OR) and 95% confidence interval (CI) were computed using the estimated coefficient during the multiple logistic regression analysis. A *p* value of <0.05 was considered statistically significant.

## Results

### Characteristics of study population

The baseline characteristics of the study population are listed in Table 1. The overall prevalence of EE was 15.9% (178 of 1,122) in men and 5.1% (40 of 780) in women. Most men with EE were classified as LA-A (157 of 178, 88.2%) and LA-B (21 of 178, 11.8%), and most women with EE were classified as LA-A (30 of 40, 75.0%) and LA-B (10 of 40, 25.0%). No subjects were classified as LA-C or LA-D. The median adiponectin levels were significantly different according to gender: 5.3 µg/ml for men and 9.3 µg/ml for women (*p*<0.001). Therefore, the data analyses were separated by gender.

In men, there were no significant differences in age, smoking, drinking status, comorbidities such as diabetes mellitus and hypertension, medication, and levels of hs-CRP, TC, HDL-C, or LDL-C between normal controls and EE groups. However, men with EE had a higher mean BMI (25.4 ± 2.6 vs 24.6 ± 2.7 kg/m^2^; *p*<0.001), waist circumference (85.7 ± 6.6 vs 83.6 ± 7.5 cm; *p*<0.001), HOMA-IR (2.1 ± 1.6 vs 1.8 ± 1.3; *p* = 0.017), TG level (158.3 ± 147.4 vs 129.5 ± 85.8 mg/dl; *p*<0.001), prevalence of metabolic syndrome (15.7% vs 10.5%; *p* = 0.043), and prevalence of hiatal hernia (12.4% vs 0.6%; *p* = 0.001). In addition, adiponectin levels were significantly lower in subjects with EE than in normal controls (median, 5.0 vs 5.4 µg/ml; *p* = 0.030).

All women was divided into post-menopausal (≥50 years old, *n* = 214, 27.4%) and pre-menopausal (<50 years old, *n* = 566, 72.6%) based on data that average age of natural menopause for Korean women is 49.7 year old. However, there were no significant differences in adiponectin levels between two groups (*p* = 0.125). In addition, there were no significant differences in any variable between normal controls and the EE groups except in the prevalence of hiatal hernia (0.3% vs 10.0%, respectively; *p*<0.001).

### Correlation between adiponectin and other clinical variables according to gender

The adiponectin level was negatively correlated with BMI, waist circumference, HOMA-IR, and TG level, but was positively correlated with the HDL-C level in both men and women. In only men, there was a weak positive correlation with age and a weak negative correlation with hs-CRP levels (Table 2).

### Association between severity of EE and indicators of obesity

**Fig. 2:**
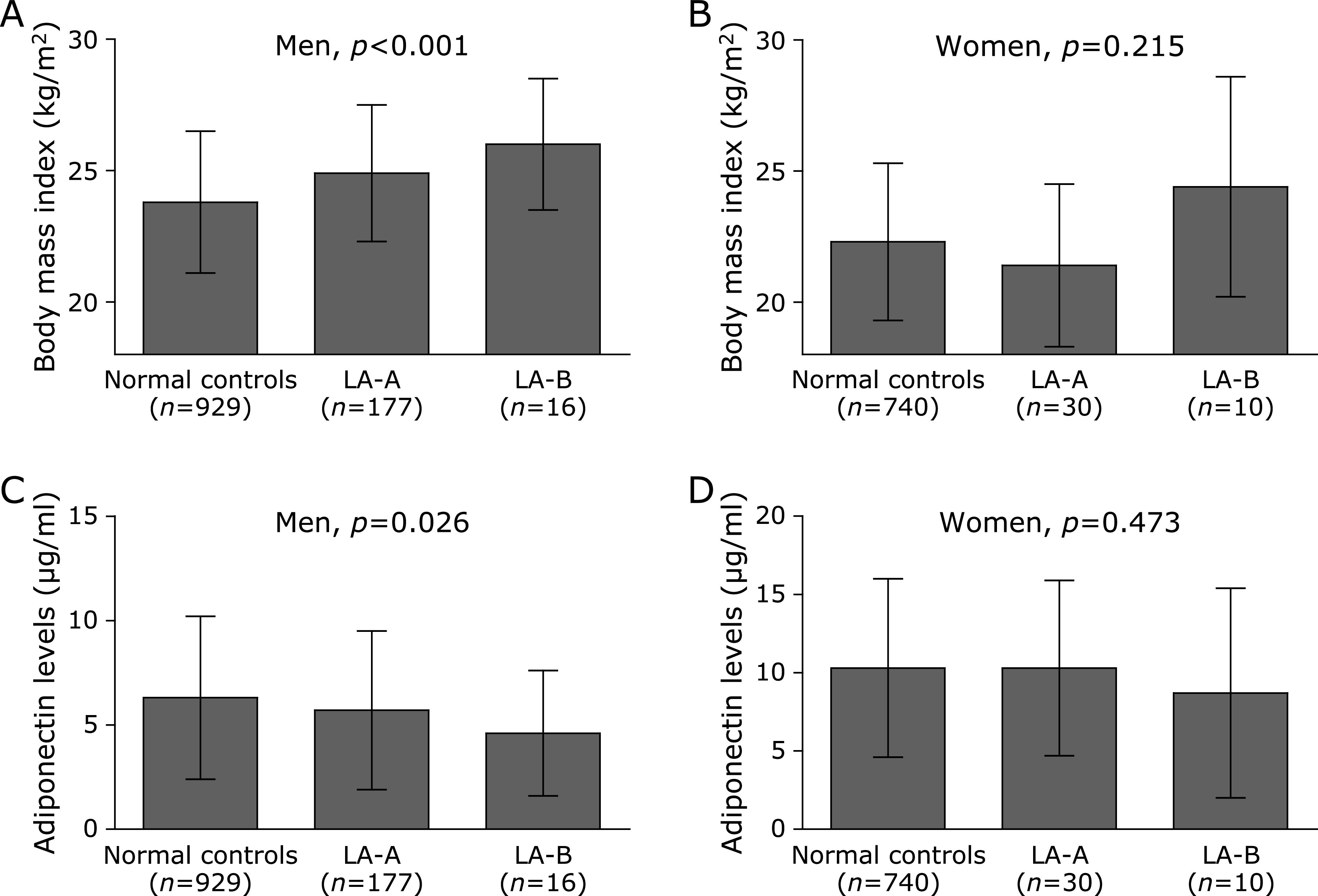
Association between severity of EE and indicators of obesity. (A) In men, BMI significantly increased as the severity of EE increased (*p*<0.001; by linear-by-linear association). (B) However, this linear association was not found in women (*p* = 0.215; by linear-by-linear association). (C) In men, adiponectin levels significantly decreased as the severity of EE increased (*p* = 0.026; by linear-by-linear association). (D) However, this linear association was not found in women (*p* = 0.473; by linear-by-linear association).

**Fig. 3:**
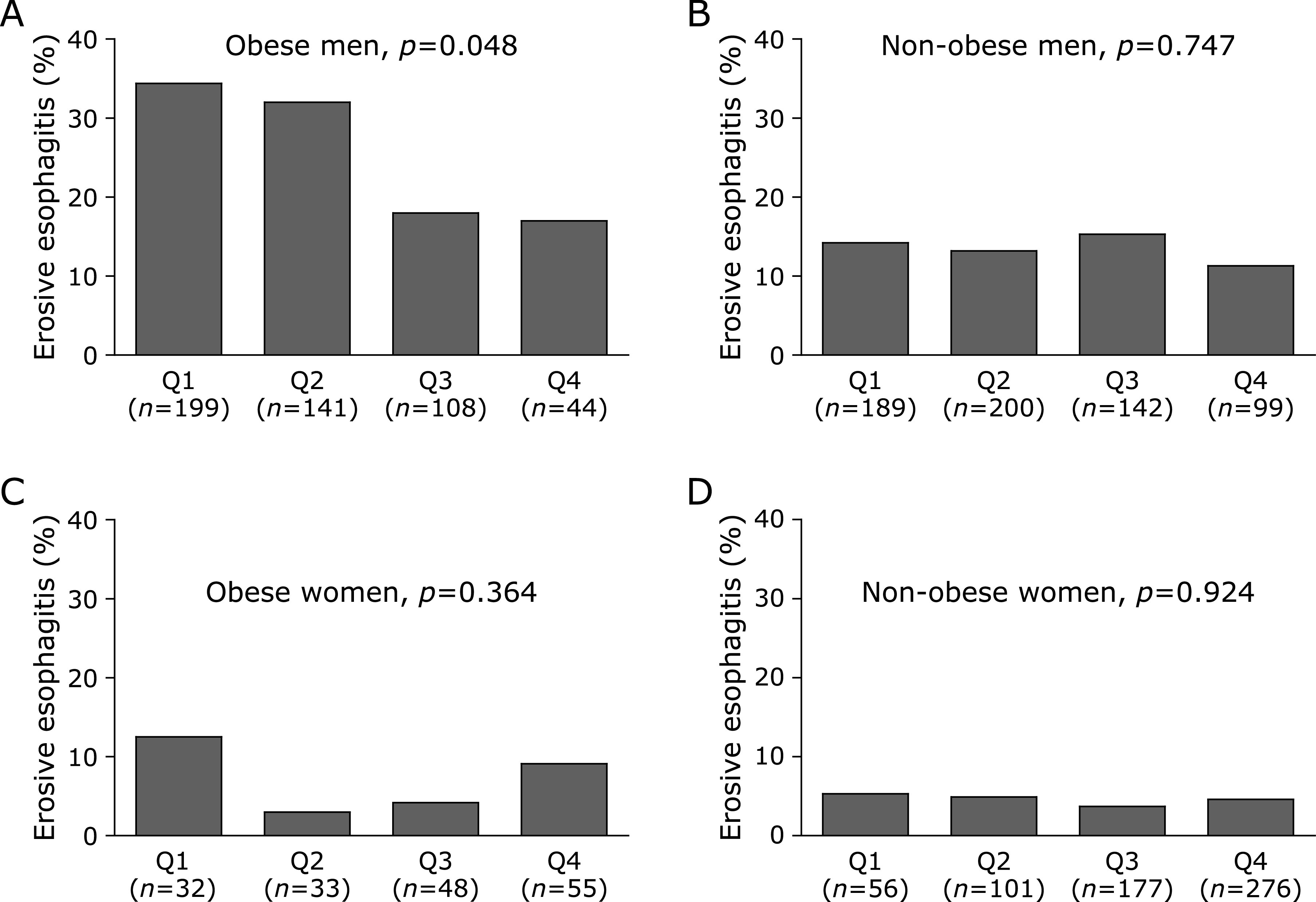
Prevalence of EE according to Qs of adiponectin levels in obese and non-obese subjects. (A) The prevalence of EE decreased proportionally with increased Qs of adiponectin levels in the obese men (*p* = 0.048; by linear-by-linear association). (B) However, this linear association was not found in non-obese men (*p* = 0.747; by linear-by-linear association). (C) In obese women, there was no linear association between prevalence of EE and Qs (*p* = 0.364; by linear-by-linear association). (D) In non-obese women, this linear association was not found (*p* = 0.924; by linear-by-linear association).

BMI significantly increased with the severity of EE in men (mean, 23.8 kg/m^2^ for normal controls, 24.9 kg/m^2^ for LA-A, and 26.0 kg/m^2^ for LA-B; *p*<0.001 by linear-by-linear association), while adiponectin levels significantly decreased (median, 6.3 µg/ml for normal controls, 5.7 µg/ml for LA-A, and 4.6 µg/ml for LA-B; *p* = 0.026 by linear-by-linear association) (Fig. 2). However, this trend was not found in women. We classified all subjects as obese or non-obese to investigate whether obesity is a major confounder in the association between adiponectin and EE. The prevalence of EE decreased proportionally with increased Qs of adiponectin levels in the obese men (34.4, 32.0, 18.0, and 17.0% for Q1–Q4 of adiponectin, respectively; *p* = 0.048 by linear-by-linear association) (Fig. 3). However, this trend was not found in non-obese men or in any women.

### Multivariate analyses of independent predictors for EE according to gender

To determine the independent predictors of EE, we performed multivariate logistic regression analyses according to gender (Table 3). The results showed that lower adiponectin levels were significantly predictors of EE in men. Using Q4 as the reference groups, Q1 (OR = 1.79, 95% CI = 1.12–2.88; *p* = 0.023) and Q2 (OR = 1.73, 95% CI = 1.08–2.78;* p* = 0.021) of the adiponectin level were significantly associated with EE. In addition, obesity (OR = 2.34, 95% CI = 1.70–3.31; *p*<0.001) and hiatal hernia (OR = 27.40, 95% CI = 10.70–70.50; *p*<0.001) were also strong predictors of EE. This was not the case for women, in whom only hiatal hernia was significantly associated with EE (OR = 35.12, 95% CI = 6.00–205.50; *p*<0.001).

### Multivariate analyses of independent predictors of obesity-associated EE

To clarity the independent predictors of obesity-associated EE, we performed the multivariate logistic regression analyses of obese and non-obese subjects (Table 4). In obese men, Q1 and Q2 of adiponectin levels showed a significantly increased OR for EE using Q4 as the reference group (OR = 1.94, 95% CI = 1.04–3.60 for Q1; OR = 2.10, 95% CI = 1.12–3.94 for Q2) after adjusting for age, hiatal hernia, and metabolic syndrome. However, lower adiponectin levels did not predict EE in non-obese men. In all of obese and non-obese men, a hiatal hernia was a strong predictor of EE (OR = 18.47, 95% CI = 2.17–157.7 in obese men; OR = 29.04, 95% CI = 10.22–82.51 in non-obese men).

## Discussion

Our results reveal the potential involvement of adiponectin in obesity-associated EE in men, thus confirming adiponectin deficiency as a humoral factor; this might be a unique characteristic in obese men with EE. This finding might also indicate that adiponectin protects against mucosal inflammation in the esophagus and that its deficiency could provoke mucosal inflammation and the development of EE in obese men.

Recent studies have shown that EE develops not only as a direct injury secondary to reflux of gastric acid into the distal esophagus, but also a result of stimulation of esophageal epithelial cells by chemokines, leaving to damage of esophageal tissues.^(25,26)^ In EE, an inflammatory response occurs by both T-cell initiation and an increase in adipose tissue-derived inflammatory mediators such as IL-6, IL-8, IL-1β, TNF-α, and leptin.^(26–29)^ Stepwise increase in the expression levels of IL-8, IL-1β, and NF-κB have been found in a wide spectrum of patients including those with normal tissue, Barrett’s epithelium, and adenocarcinoma.^(26)^ Decreased levels of serum adiponectin were shown in patients with severe GERD evaluated by viodeoesophagography and these adiponectin levels correlated inversely with BMI.^(18)^ With respect to the anti-inflammatory role of adiponectin, studies have shown that adiponectin levels are negatively associated with several inflammatory markers including IL-6 and CRP, but positively associated with some anti-inflammatory markers such as IL-10.^(30–32)^ Previous studies have also shown that hs-CRP levels are highly correlated with levels of high molecular weight adiponectin.^(33,34)^ Hs-CRP is an acute-phase marker of inflammation in cardiovascular events or metabolic syndrome.^(28,35)^ Therefore, we checked the hs-CRP level as another mediator of inflammation in EE. Although there was a reciprocal association between adiponectin and hs-CRP, the correlation was weak and only present in men. The reason might be that most subjects had a mild form of EE, therefore, they did not develop an increase in systemic hs-CRP levels.

In theory, to inhibit esophageal inflammation, adiponectin secreted from visceral adipose tissues should bind to two types of adiponectin receptors in the esophagus: AdipoR1 and AdipoR2. Studies have demonstrated the presence of AdipoR1 and AdipoR2 in both Barrett’s esophagus and normal esophagus.^(36,37)^ It was confirmed that adiponectin receptors were downregulated in Barrett’s esophagus compared to normal squamous epithelium from the same patients. In addition, lower expression of adiponectin receptors was observed in morbidly obese controls.^(37)^ Therefore, obesity-associated stimuli might regulate the expression of adiponectin receptors as well. However, given the limited number of studies, little conclusive evidence exists regarding adiponectin receptors in obesity-associated EE. In addition, we confirmed the strong relationship between GERD and mechanical change, which has traditionally been considered to have a role in pathophysiology of GERD. In men, OR of hiatal hernia for EE was relatively higher than that of adiponectin. In women, hiatal hernia remained the strong risk factor for EE. Adiponectin was not risk factor for EE in women. Therefore, serum adiponectin level might play a minor role in the development of EE, not major role alike hiatal hernia.

Considering the role of adiponectin in pathophysiology of EE, either adiponectin supplement or various dietary components increasing the concentration of adiponectin could possibly provide the novel treatment option for proton pump inhibitor-refractory GERD. Based on our results, we could expect to achieve greater effect among obese men with EE. So far, there was no study whether either adiponectin supplement or various dietary components increasing the concentration of adiponectin improve the EE or not. However, several epidemiologic and intervention studies have demonstrated the increasing adiponectin concentration after the intake of polyunsaturated fat, eicosapentaenoic acid, and ω-3 supplements.^(38–42)^ Further researches about the efficacy of adiponectin supplements or various dietary components increasing the concentration of adiponectin on EE seem to be needed.

We identified gender-specific differences in the adiponectin levels in EE. One study that reported decreased adiponectin levels in patients with EE only included men, and other studies have also reported significant negative correlations of adiponectin levels in small samples and mixed-gender populations with EE.^(19,20)^ In the present study, we stratified the analyses by gender and found strikingly different patterns of association. Normal adiponectin levels of women were significantly higher than those of men; additionally, the levels were significantly different among patients with EE according to gender, decreasing with an increase in EE severity only in men. This gender-dependent difference in adiponectin has also been observed in metabolic syndrome, diabetes mellitus, anxiety disorder, sleep disorder, and cardiovascular disease.^(43–46)^ This difference could be due the higher levels of high-molecular-weight adiponectin in women, the gender-specific haplotype of the adiponectin gene, or the different fat distributions of men and women; men trend to have a central fat distribution, while women tend to have a peripheral fat distribution.^(47–49)^ Other data support the idea that testosterone has a direct suppressive effect on adiponectin secretion.^(50)^ For example, deceases in testosterone with castration are associated with high adiponectin levels. Testosterone treatment in castrated mice also reduces adiponectin levels.^(51)^ Similarly, a study included both menopause women and old men demonstrated that gender-hormone such as testosterone and bioavailable estradiol concentration was each associated with adiponectin. This gender hormone–adiponectin association was true for both men and women after adjusting multiple variables.^(51,52)^ However, before discussing these gender-differences of adiponectin levels, we should confirm enough samples in both gender for generalization of gender-difference in adiponectin levels. In our study, the population size of women was smaller than that of men. The underlying mechanisms of the gender-related differences in adiponectin levels require further investigation, and the implications of these mechanisms in the initiation and progression of EE must be better characterized in men and women.

This study had several strengths. First, GERD itself is a heterogeneous disease entity, and various risk factors such as obesity, gender, smoking, and alcohol use might function as confounders. Therefore, we enrolled homogeneous subjects with endoscopically diagnosed EE. We also considered various risk factors in our data analyses. Second, previous studies had poor power because of small sample sizes. However, we had higher power because of our larger sample. Third, adiponectin level shows a skewed deviation, and the normal ranges varied according to gender. Therefore, we used the log-transformed values of adiponectin and stratified them by gender. This may be one of the most important points of GERD and adiponectin studies.

However, there were also some limitations. First, the subjects were obtained from a health-screening program. Therefore, most subjects had mild EE, and the impact of adiponectin on obesity-associated EE might have been underestimated. Second, there are potential confounders at different levels of the causal chains linking obesity to GERD, including mechanical determinants, induvial behavioral factors, hormonal and metabolic disturbances. However, we didn’t full adjustment for potential confounders due to our cross-sectional design. Therefore, we can’t exclude the possibility that adiponectin is a simple innocent bystander for EE. Prospective studies are needed with appropriate control of potential confounding variables.

In conclusions, the present study demonstrated the potential link between adiponectin and obesity-associated EE. Our data suggests that not only mechanical cases generally accepted as main role for the development of GERD, but also humoral changes such as adiponectin levels might be partially related to development of EE. Our data suggests that underlying mechanisms of EE may be related not only to mechanical causes but also to humoral causes such as adiponectin levels. However, how adiponectin signaling regulates inflammation in EE remains unclear. A better understanding of this mechanism might also be helpful to prevent EE and progression of the complications of EE, such as Barrett’s esophagus and esophageal adenocarcinoma.

## Author Contributions

HKJ designed and integrated the research. HKJ and SHJ were involved in patient recruitment, and gaining ethical approval. HKJ and CHT were involved in analyses and interpretation of data. CHT wrote the first draft of the manuscript. All authors reviewed, edited the manuscript, and approved the final version of the manuscript.

## Figures and Tables

**Table 1:** Baseline characteristics of subjects according to gender

	Men (*n* = 1,122)				Women (*n* = 780)		
	Normal controls (*n* = 944)	Erosive esophagitis (*n* = 178)	*p* value		Normal controls (*n* = 740)	Erosive esophagitis (*n* = 40)	*p* value
Age (years), mean ± SD	45.6 ± 9.4	45.5 ± 9.0	0.803		44.4 ± 9.7	47.3 ± 11.6	0.068
BMI (kg/m^2^), mean ± SD	24.6 ± 2.7	25.4 ± 2.6	<0.001		22.8 ± 0.1	23.1 ± 0.6	0.533
<25.0, *n* (%)	558 (59.1)	72 (40.4)	<0.001		583 (78.8)	28 (70.0)	0.234
≥25.0, *n* (%)	386 (40.9)	106 (59.6)			157 (21.2)	12 (30.0)	
Waist circumference (cm), mean ± SD	83.6 ± 7.5	85.7 ± 6.6	<0.001		75.9 ± 33.8	75.5 ± 7.7	0.937
Adiponectin ( µg/ml)							
Mean ± SD	6.3 ± 4.0	5.7 ± 2.9	0.008		10.3 ± 5.7	10.2 ± 5.6	0.395
Median (min–max)	5.4 (0.5–37.0)	5.0 (0.2–15.8)	0.03		9.3 (0.5–0.3)	9.1 (0.5–0.8)	0.708
HOMA-IR, mean ± SD	1.8 ± 1.3	2.1 ± 1.6	0.017		16.2 ± 10.7	15.4 ± 8.6	0.65
Hs-CRP (mg/dl), mean ± SD	0.1 ± 0.2	0.1 ± 0.2	0.909		0.1 ± 0.1	0.2 ± 0.1	0.875
TC (mg/dl), mean ± SD	192.1 ± 35.9	192.1 ± 32.2	0.908		184.5 ± 32.7	190.8 ± 36.0	0.235
TG (mg/dl), mean ± SD	129.5 ± 85.8	158.3 ± 147.4	<0.001		80.2 ± 45.0	89.6 ± 50.3	0.198
HDL-C (mg/dl), mean ± SD	49.1 ± 10.8	48.6 ± 12.0	0.539		58.4 ± 12.2	58.3 ± 14.2	0.967
LDL-C (mg/dl), mean ± SD	107.7 ± 27.2	104.0 ± 28.2	0.098		99.9 ± 22.3	102.6 ± 32.7	0.576
Current smoker, *n* (%)	140 (14.8)	32(18.0)	0.252		34 (4.6)	2 (5.0)	0.756
Heavy drinking, *n* (%)	553 (58.6)	102 (57.3)	0.725		108 (14.5)	5 (12.5)	0.223
Metabolic syndrome, *n* (%)	99 (10.5)	28 (15.7)	0.043		34 (4.6)	2 (5.0)	0.707
Hypertension, *n* (%)	164 (17.4)	27 (15.2)	0.389		181 (24.5)	15 (37.5)	0.064
Diabetes mellitus, *n* (%)	189 (20.0)	28 (15.7)	0.57		16 (2.2)	1 (2.5)	0.595
Medication, *n*(%)							
NSAID	40 (4.3)	15 (7.8)	0.064		38 (5.1)	2 (5.0)	0.553
PPI or H2B*	16 (1.8)	0	0.09		10 (1.5)	2 (6.9)	0.085
Statin*	37 (4.1)	8 (4.5)	0.802		22 (3.3)	1 (3.4)	0.961
Thiazolidinedione*	0	1 (0.6)	0.164		2 (0.3)	0	1
Hiatal hernia, *n* (%)	6 (0.6)	22 (12.4)	0.001		2 (0.3)	4 (10.0)	<0.001
Erosive esophagitis, *n*(%)							
LA-A		157 (88.2)				30 (75.0)	
LA-B		21 (11.8)				10 (25.0)	

BMI, body mass index; HOMA-IR, Homeostasis model assessment of insulin resistance; hs-CRP, high-sensitivity C reactive protein; TC, total cholesterol; TG, triglyceride; HDL-C, high-density lipoprotein cholesterol; LDL-C, low-density lipoprotein cholesterol; NSAID, nonsteroidal anti-inflammatory drug; PPI, proton pump inhibitor; H2B, histamine 2 blocker; LA, Los Angeles Classification. *Medication history about PPI or H2B, statin, and thiazolidinedione was missing in 127 (6.7%) subjects.

**Table 2:** Correlation between adiponectin and other clinical variables

	Men (*n* = 1,122)			Women (*n* = 780)	
	*r*	*p* value		*r*	*p* value
Age	0.078	0.009		–0.009	0.78
BMI	–0.183	<0.001		–0.162	<0.001
Waist circumference	–0.183	<0.001		–0.17	<0.001
HOMA-IR	–0.211	<0.001		–0.181	<0.001
Hs-CRP	–0.102	0.005		–0.078	0.066
TC	–0.047	0.116		0.059	0.999
TG	–0.234	<0.001		–0.19	<0.001
HDL-C	0.207	<0.001		0.289	<0.001
LDL-C	–0.015	0.624		–0.043	0.23

BMI, body mass index; HOMA-IR, Homeostasis model assessment of insulin resistance; hs-CRP, high-sensitivity C reactive protein; TC, total cholesterol; TG, triglyceride; HDL-C, high-density lipoprotein cholesterol; LDL-C, low-density lipoprotein cholesterol.

**Table 3:** Multivariate analyses of independent predictors for erosive esophagitis

Variables	Odds ratio	95% confidence interval	*p* value
Men			
Adiponectin			
Q1	1.79	1.12–2.88	0.023
Q2	1.73	1.08–2.78	0.021
Q3	1.49	0.91–2.43	0.11
Q4	1.00 (reference)	—	—
Obesity	2.34	1.70–3.31	<0.001
Hiatal hernia	27.4	10.70–70.50	<0.001
Metabolic syndrome	1.16	0.72–1.85	0.532

Women			
Adiponectin			
Q1	0.99	0.32–3.14	0.995
Q2	1.69	0.73–3.91	0.223
Q3	0.86	0.37–2.02	0.722
Q4	1.00 (reference)	—	—
Obesity	1.41	0.65–3.02	0.384
Hiatal hernia	35.12	6.00–205.50	<0.001
Metabolic syndrome	0.93	0.20–4.27	0.921

Qs, quartiles of adiponectin level.

**Table 4:** Multiple logistic regression analyses of predictors for erosive esophagitis in obese and non-obese subjects

Model	Variables	Odds ratio	95% confidence interval	*p* value
Men				
Obese	Adiponectin			
	Q1	1.94	1.04–3.60	0.036
	Q2	2.1	1.12–3.94	0.021
	Q3	1.28	0.64–2.56	0.486
	Q4	1.00 (reference)		—
	Hiatal hernia	18.47	2.17–157.7	0.008
	Metabolic syndrome	1.3	0.77–2.19	0.323
Non-obese	Adiponectin			
	Q1	1.65	0.78–3.45	0.188
	Q2	1.31	0.63–2.75	0.468
	Q3	1.72	0.86–3.42	0.124
	Q4	1.00 (reference)		—
	Hiatal hernia	29.04	10.22–82.51	<0.001
	Metabolic syndrome	0.68	0.20–2.28	0.527

Women				
Obese	Adiponectin			
	Q1	2.57	0.56–11.70	0.224
	Q2	0.28	0.02–3.53	0.322
	Q3	0.61	0.10–3.62	0.587
	Q4	1.00 (reference)		—
	Hiatal hernia	41.49	2.54–677.35	0.009
	Metabolic syndrome	0	0	0.998
Non-obese	Adiponectin			
	Q1	0	0	0.989
	Q2	2.36	0.94–5.97	0.068
	Q3	0.93	0.34–2.50	0.88
	Q4	1.00 (reference)		—
	Hiatal hernia	41.28	3.46–492.82	0.003
	Metabolic syndrome	3.75	0.74–19.16	0.112

Qs, quartiles of adiponectin level.
